# Case Report: Successful surgical management of a challenging primary cardiac angiosarcoma

**DOI:** 10.3389/fcvm.2023.1279177

**Published:** 2023-11-03

**Authors:** Yichen Li, Yinze Ai, Weijie Tang, Jijia Liu, Jinfu Yang, Chengming Fan

**Affiliations:** Department of Cardiovascular Surgery, the Second Xiangya Hospital, Central South University, Changsha, China

**Keywords:** primary cardiac angiosarcoma, cardiac surgery, malignant, reconstruction, SVC

## Abstract

Primary cardiac tumors are exceptionally rare, with malignant tumor occurrences ranging from 0.0017% to 0.28%. Among these, primary cardiac angiosarcoma (PCA) stands as the most prevalent malignancy, primarily impacting the right cardiac system. In this case report, we present the instance of a 44-year-old woman who recently exhibited acute chest discomfort and was subsequently diagnosed with a microangiosarcoma within the right atrium and superior vena cava. Diagnostic modalities including chest x-rays, CT, MRI, and PET-CT were instrumental in pinpointing the tumor's location and nature. Surgical excision followed by pathological and immunological examinations confirmed the diagnosis. The patient's recovery post-surgery has been encouraging, with successful follow-up chemoradiotherapy administered. Despite advancements, devising optimal strategies for enhancing patient survival and quality of life in angiosarcoma cases remains a pressing research challenge.

## Introduction

Primary cardiac tumors are infrequently encountered, with a subset manifesting as malignant tumors at an incidence ranging from 0.0017% to 0.28% (1). Among these, benign tumors constitute 75%, encompassing cardiac myxoma, rhabdomyoma, fibroma, hemangioma, and teratoma. Myxoma, for instance, predominantly arises in the left atrium. Conversely, 25% of primary cardiac tumors exhibit malignancy, including sarcoma, lymphoma, and mesothelioma. The spotlight, however, is on primary cardiac angiosarcoma (PCA), the foremost malignant cardiac neoplasm. PCA predominantly affects the right cardiac system and constitutes the majority of malignant cardiac tumors. While radical excision remains the primary therapeutic approach, the unfortunate reality is that a staggering 89% of patients present with metastases at diagnosis (2, 3). Despite multidisciplinary interventions such as radiation, chemotherapy, and targeted therapy, the prognosis remains grim, with surgically treated patients reporting a median survival of merely 14 months (4). As a consequence, these patients often receive chemotherapy and radiation therapy, with the lack of standardized treatment protocols posing a persistent challenge. Notably, the current chemotherapy regimens are extrapolated from extracardiac soft tissue sarcoma data, with doxorubicin and ifosfamide being prevalent choices (5). Even though some similar cases have been reported, in this report, we detail the case of a 44-year-old woman diagnosed with a substantial PCA within the right atrium and superior vena cava and successful surgical management with beating heart technique.

## Case report

**Figure 1:**
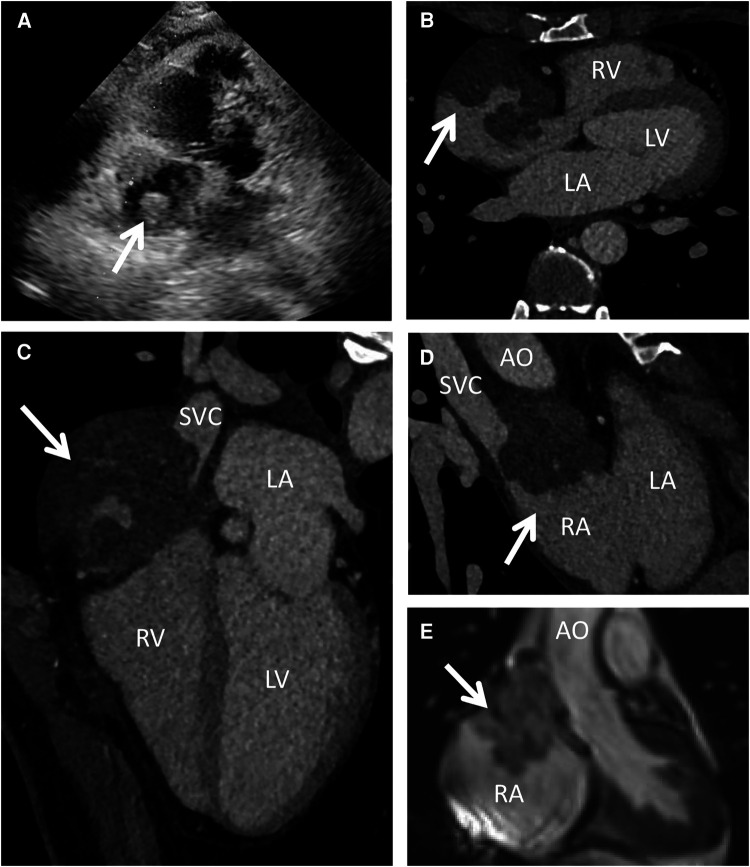
Preoperative echocardiography, cardiac CT and MRI. Echocardiography (**A**) showed multiple mixed echogenic masses on the lateral wall of the right atrium; CT scan (**B–D**) showed a giant cardiac mass with the invasion of the RA and SVC; MRI (**E**) indicated that the mass attached to the wall of RA with unclear boundary. AO, ascending aorta; LA, left atrium; LV, left ventricle; RA, right atrium; RV, right ventricle; SVC, superior vena cava.

A 44-year-old woman presented with sudden chest discomfort of unknown origin, prompting a comprehensive medical examination conducted one month prior. This examination revealed the presence of a mass in her right atrium. The patient's chest discomfort occurred without accompanying symptoms such as shortness of breath, coughing, or other pulmonary issues. These symptoms typically subsided after a brief 2-minute rest. Through thorough patient interviews, we established the absence of significant medical history and a lack of familial occurrence of similar medical conditions. Notably, prior to admission, a thorough evaluation had unveiled a substantial pericardial effusion, promptly addressed via pericardiocentesis upon admission. No significant abnormal findings were observed in the cytology of the pericardial fluid. Subsequently, a battery of diagnostic procedures was conducted post-admission, encompassing a chest x-ray, cardiac ultrasound, electrocardiogram, cardiac CT, cardiac MRA, and PET-CT. The patient exhibited a normal electrocardiogram indicative of sinus rhythm. Preoperative echocardiography indicated an enlarged heart shadow, delineating multiple masses of uncertain nature in the right atrium, including a minor pericardial effusion (Figure 1A). The cardiac CT highlighted a right atrium lesion with superior vena cava invasion (Figures 1B–D). The cardiac MRA was performed and depicted an occupying lesion in the right atrium, signifying a high likelihood of a malignant tumor (Figure 1E). This assessment was further confirmed by PET-CT findings of multiple nodules with significantly elevated glucose metabolism in the right atrium and concurrent pericardial effusion.

**Figure 2:**
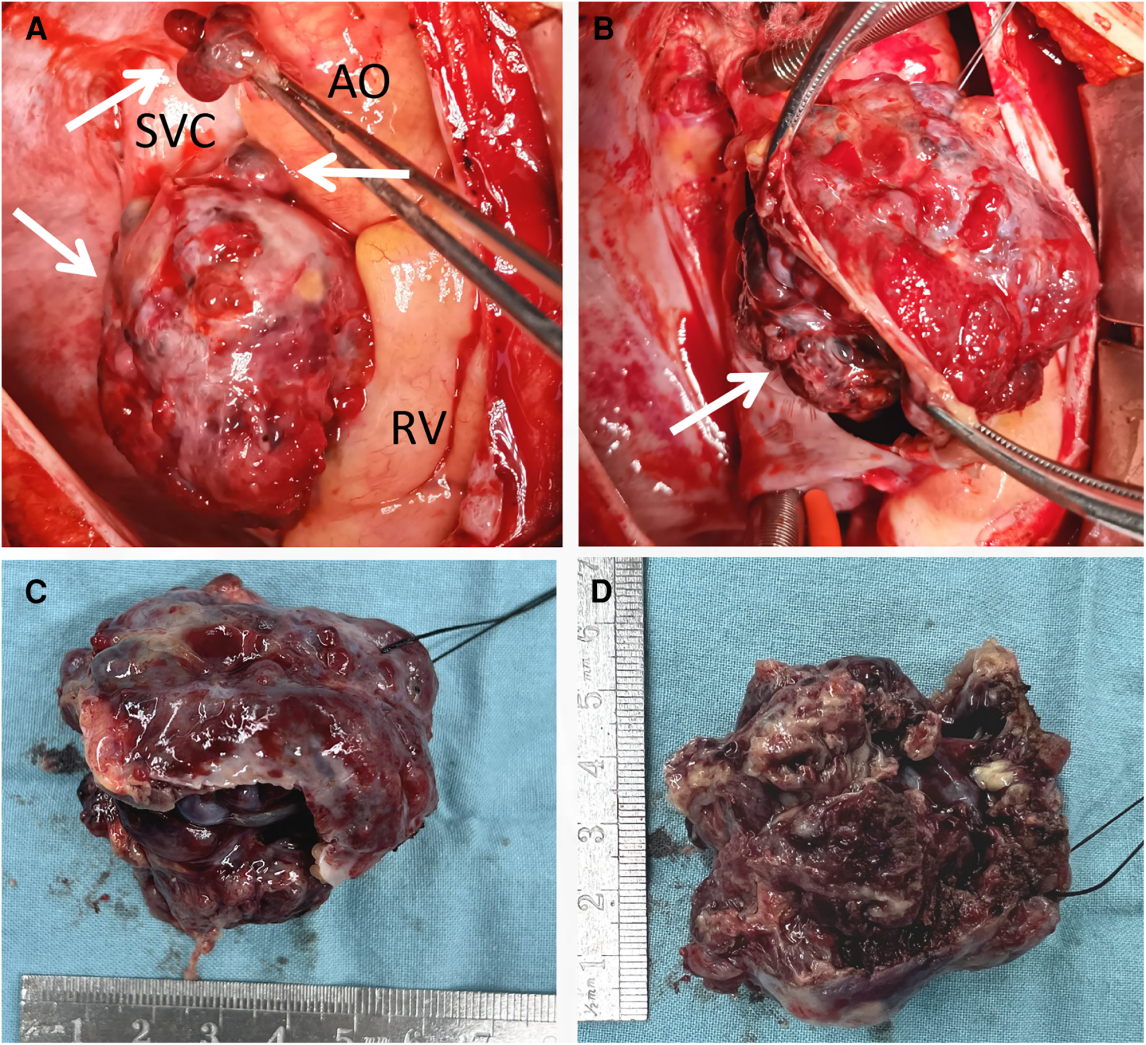
Intraoperative view showing a giant mass located in the RA, SVC and pera-AO (**A**), infiltrating the wall of RA and SVC (**B**); after totally removal, the mass was measured approximately 5 cm × 5 cm × 4 cm, exhibited parenchymal characteristics, a firm consistency, dark red coloration, and an uneven surface (**C,D**). AO, ascending aorta; RV, right ventricle; SVC, superior vena cava.

**Figure 3:**
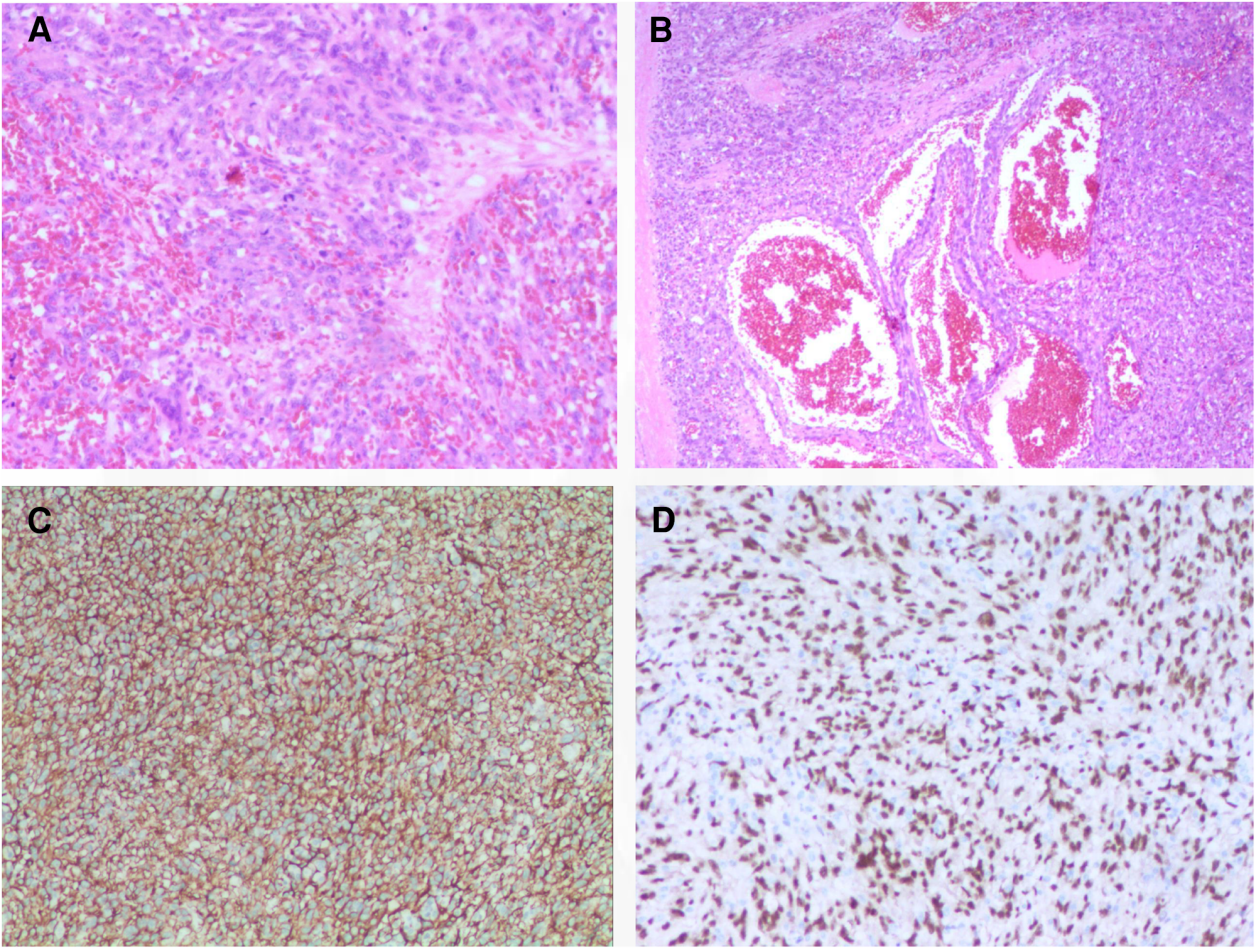
Histology and immunohistochemistry of the masses. Malignant tumor derived from mesenchymal tissue, with obvious cell atypia, many mitoses, large areas of hemorrhage and necrosis, rich blood vessels, invasion of surrounding muscle tissue with HE staining (**A,B**); immunohistochemical support for angiosarcoma with positive of CD34 (**C**), ERG (**D**), et al. 100× for panel A, C and D; 40× for panel B.

**Figure 4:**
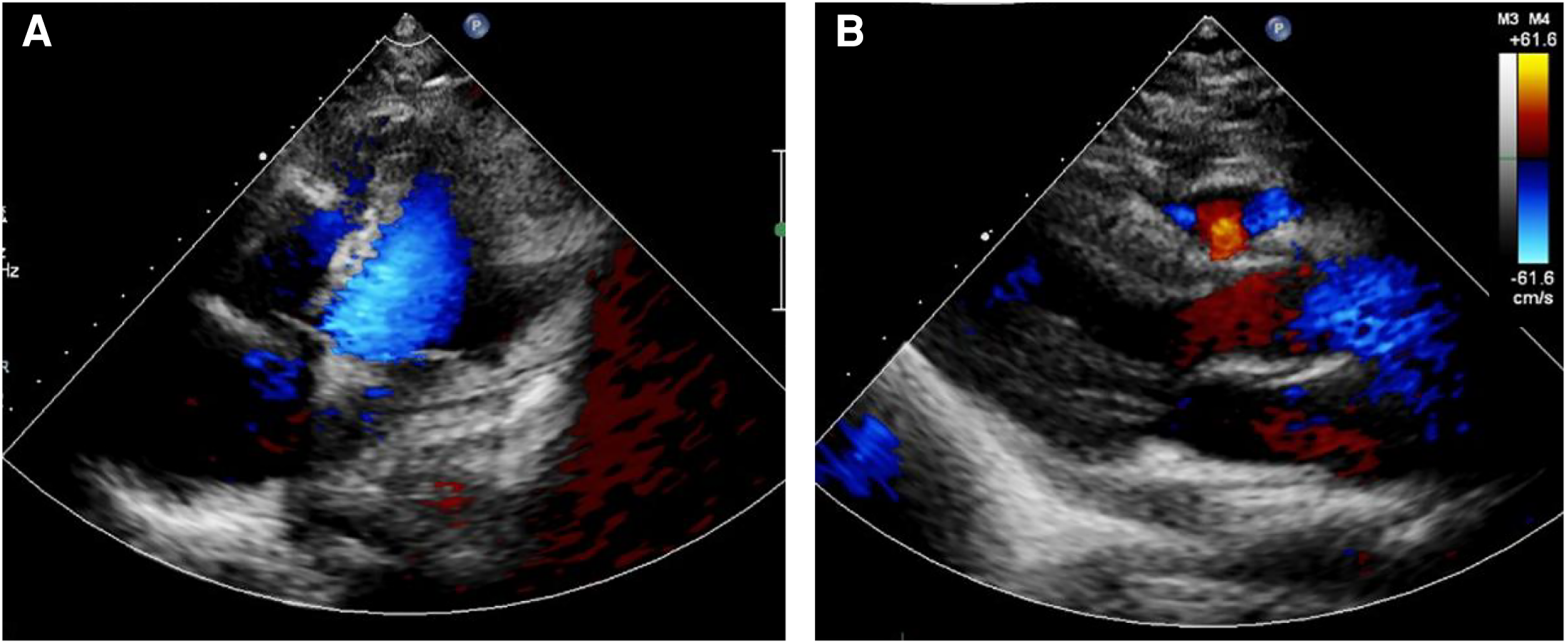
Postoperative echocardiography showed that the right atrial mass was totally resected (**A**), no hemodynamic abnormality (**B**) and no recurrence signals.

Following the comprehensive evaluation, immediate surgical resection of the cardiac tumor was performed. A giant mass was observed extent encompassing from the right atrial appendage and the superior vena cava, to the right atrioventricular sulcus, the posterior atrial wall, and approximately 2 cm downward from the inferior vena cava opening (Figure 2A, Supplementary Video S1). A cluster of three masses, totaling about 3 cm in diameter, was found located on the right side of the aorta. Notably, a pale red coloration marked the pericardial fluid. An incision was made in the right atrium, facilitating the removal of the mass and the invaded right atrial wall (Figure 2B, Supplementary Video S1). During surgery, the tumor, a small margin (2 cm–3 cm) of the surrounding healthy tissue was removed. Because the procedure was performed with beating heart, tumor excision was performed with great care to avoid damaging the sinus node and causing complete atrioventricular block. The mass, measuring approximately 5 cm × 5 cm × 4 cm, exhibited parenchymal characteristics, a firm consistency, dark red coloration, and an uneven surface (Figures 2C,D). The right atrium was finally reconstructed using a bovine pericardium patch of matching size. The pathology postoperatively from both the right atrium and para-aorta were detected similar and revealed that the grayish-red solid mass featured a 2.5 cm × 2 cm × 1.5 cm size portion showcasing pronounced cellular heterogeneity, conspicuous nuclear divisions, extensive hemorrhagic necrosis, and abundant blood vessels (Figures 3A,B). Immunohistochemistry findings for the right atrial cardiac adenoma indicated CK (-), S100 (-), TLE1 (-), Ki67 (80%+), P53 (5% weak+, possibly indicative of nonsense mutant type expression), Desmin (-), MyoD1 (-), and Miyogenin (-). Immunophenotypic markers SMA (weak+), CD34 (++), CD31 (++), F8 (++), D2-40 (-), ERG (++), HCAL (-), ER (O), P16 (focal+), and CD10 (-) were documented (Figures 3C,D). Post-surgery, the patient's stay in the intensive care unit spanned 18.5 h and discharge on the 12th day with the recommendation of transferred to the oncology department and subsequently underwent conventional and experimental chemoradiotherapy for angiosarcoma [stereotactic radiotherapy and weekly paclitaxel (80–100 mg/m^2^) for 6 cycles]. During the ensuing six-month follow-up period, the patient exhibited a favorable recovery trajectory devoid of complications, ultimately experiencing a resumption of normal activities without the recurrence of symptoms. The postoperative echocardiography revealed that the right atrial mass was totally resected, no hemodynamic abnormality and no recurrence signals (Figures 4A,B).

## Discussion

Angiosarcoma, a highly malignant endothelial cell tumor, constitutes 1%–2% of soft tissue tumors. It manifests as an exceptionally aggressive cancer with a bleak prognosis, capable of affecting any anatomical region (3). Notably, around 60% of lesions are identified within the epidermis, soft tissues, liver, spleen, bones, and breast. Contrastingly, the heart and kidneys exhibit lower incidence rates (6). PCA stands as a predominant form of primary cardiac malignancy, with 90% of cases arising in the right atrium, while the left atrium or ventricle accounts for less than 5% (4, 7). PCA typically emerges in male patients aged 30–40 (8), and its clinical symptoms are non-specific, contingent upon location and size. The swift infiltrative growth of the tumor results in acute clinical manifestations, including chest pain, constriction, shortness of breath, arrhythmia, pericardial effusion, and right heart failure (9, 10). Given its aggressive nature, the prognosis of PCA is dire, attributed to high local recurrence and early metastasis rates at diagnosis (11, 12).

### Imaging techniques and diagnosis

Diagnostic techniques for primary cardiac lesions encompass x-ray, echocardiography, CT, MRI, and PET-CT, with CT and MRI being pivotal for diagnosis (13). Echocardiography has evolved as a primary imaging method due to its precision in tumor size and location assessment, evaluating heart valves, pericardial structures, and its non-invasive, cost-effective nature. However, limitations exist, including operator dependence and constrained visual fields in specific patients (14). CT, especially for lung tissue characterization and systemic metastasis, is more effective. Contrast-enhanced CT scans offer valuable insights into tumor properties, blood vessels, heart walls, and lung parenchyma, minimizing the likelihood of missing lesions (15). On CT scans, PCA showcases uniform or asymmetrical density on unenhanced images, whereas enhanced images exhibit heterogeneous centripetal enhancement (16). Cardiac MRI is regarded as an advanced technique for determining tumor size, location, and signal characteristics, distinguishing between solid, liquid, blood, and fat (17). Additionally, PET-CT aids in discerning benign from malignant tumors, assessing invasiveness, and identifying metastasis. Immunohistochemical markers like CD31, CD34, ERG, and factor VIII confirm PCA's endothelial cell origin (18). The patient's thorough examinations indicated the likelihood of angiosarcoma, requiring essential tissue biopsy for confirmation. Following tumor resection, pathological and immune examinations were conducted, revealing both angiosarcoma and angiomyxfibroma derived from mesenchymal tissue.

### Treatment approaches and outlook

Due to the rarity of these tumors, optimal management strategies remain under debate. Presently, available treatments encompass surgery, radiation therapy, and chemotherapy. Surgical excision remains the preferred approach for primary malignant cardiac tumors, yielding the best potential for long-term survival and curative outcomes (19, 20). Median survival for primary cardiac malignancies ranges from 6 to 11 months, while surgical patients experience a median survival of 14 months compared to 3.8 ± 2.5 months for those with inoperable metastases (21, 22). Combining surgical excision and chemoradiotherapy enhances short-term prognosis but lacks discernible effects on long-term outcomes (23). Heart transplantation is an option when resection is unfeasible due to metastases, though prolonged immunosuppressant use post-surgery may induce recurrence and metastasis. Radiation therapy, particularly stereotactic body radiotherapy (SBRT), has yielded positive results with tumor regression and minimal long-term effects (24). Investigation into targeted drugs and immunotherapy shows promise, particularly taxanes as adjuvant chemotherapy for cardiac angiosarcoma (25). Although the optimum chemotherapy regimen is yet to be clearly defined, molecularly targeted medications like amlotinib, imatinib, sorafenib, and bevacizumab hold therapeutic potential (26, 27). The patient's history underscores the significance of swift surgical intervention in cases like a massive right atrial angiosarcoma. The current recovery of the patients is highly encouraging following systematic anti-tumor chemoradiotherapy post-surgery. The present case reveals that the disease had progressed to an advanced stage of angiosarcoma without presenting any discernible symptoms. This serves as a stark reminder of the dangers associated with this condition. The present case underscores the critical importance of swift diagnosis and surgical intervention when dealing with right atrial angiosarcoma. Surgical excision, when combined with subsequent chemoradiotherapy, continues to hold promise as an approach to manage this rare and aggressive cardiac malignancy. Further research and investigation are imperative to optimize diagnostic and therapeutic strategies, ultimately leading to improved patient outcomes.

## Conclusion

In conclusion, primary cardiac angiosarcoma, especially within the right atrium, represents an unusual clinical entity. While various imaging modalities aid in diagnosis, treatment strategies for PCA remain elusive, necessitating prompt interventions post-surgical resection. Given the short time follow-up period and rarity of this malignancy, the challenges it poses underscore the imperative of ongoing research to unravel improved diagnostic and therapeutic approaches.

## Data Availability

The raw data supporting the conclusions of this article will be made available by the authors, without undue reservation.
